# HHV-6 Specific T-Cell Immunity in Healthy Children and Adolescents

**DOI:** 10.3389/fped.2018.00191

**Published:** 2018-07-02

**Authors:** Christine M. Schwarz, Volker Strenger, Heimo Strohmaier, Georg Singer, Margarita Kaiser, Andrea Raicht, Wolfgang Schwinger, Christian Urban

**Affiliations:** ^1^Division of Pediatric Hematology/Oncology, Department of Pediatrics and Adolescent Medicine, Medical University of Graz, Graz, Austria; ^2^Center for Medical Research (ZMF), Core Facility Flow Cytometry, Medical University of Graz, Graz, Austria; ^3^Department of Pediatric and Adolescent Surgery, Medical University of Graz, Graz, Austria

**Keywords:** HHV-6, T-cell response, flow cytometry, U54, antigen-specific T-cells

## Abstract

**Objective:** Primary infection with human herpes virus 6 (mainly HHV-6B) commonly occurs in the first 2 years of life leading to persistence and the possibility of virus reactivation later in life. Consequently, a specific cellular immune response is essential for effective control of virus reactivation. We have studied cell-mediated immune response to HHV-6 (U54) in healthy children and adolescents.

**Materials and Methods:** By flow cytometry, the amount of cytokine (interferon gamma—IFN- γ, interleukin 2—IL-2, tumor necrosis factor alpha—TNF-α) secreting T-cells were measured after 10 days of pre-sensitization and 6 h of re-stimulation with mixtures of pooled overlapping peptides from U54, staphylococcal enterotoxin B (SEB, positive control), or Actin (negative control) in healthy children and adolescents without any underlying immune disorder or infectious disease.

**Results:** All individuals showed a virus-specific response for at least one cytokine in either CD4+ or CD8+ cells. Percentages of individuals with HHV-6-specific TNF-α response in CD4+ (48% of individuals) as well as CD8+ (56% of individuals) were always the highest. Our data show significantly higher frequencies of HHV-6-specific TNF-α producing CD8+ T-cells in individuals older than 10 years of life (*p* = 0.033). Additionally, the frequency of HHV-6 specific TNF-α producing CD8+ T-cells positively correlated with the age of the individuals. Linear regression analysis showed a positive relation between age and frequency of HHV-6-specific TNF-α producing CD8+ T-cells.

**Conclusion:** Results indicate that T-cell immune response against HHV-6 is commonly detectable in healthy children and adolescents with higher frequencies of antigen-specific T-cells in older children and adolescents possibly reflecting repeated stimulation by viral persistence and subclinical reactivation.

## Introduction

Human herpesvirus-6 (type HHV-6A) was isolated for the first time in 1986 from patients with lymphoproliferative disorders (1). The clinically more relevant species HHV-6B is known as a causative agent of exanthema subitum (2).

Primary infection with HHV-6B occurs within the first 2 years of life and is often associated with exanthema subitum (roseola) (3, 4). In most regions of the world, seroprevalence of HHV-6 ranges up to 100% (5). Like other herpes viruses, the virus persists in the host after primary infection (latent infection) and may reactivate under immunosuppression (6). In healthy individuals, HHV-6 reactivation usually occurs without significant symptoms, but in immunocompromised subjects such as solid organ or hematopoietic stem cell transplant recipients, reactivation can lead to CNS morbidity, pneumonitis, transplant rejection, or delayed engraftment (6, 7). In addition, an inherited chromosomal integration of HHV-6 DNA (ciHHV-6) into the human genome has been described leading to high DNA copy numbers by using quantitative PCR. Despite an estimated prevalence of 0.2–5%, the clinical impact of this phenomenon still remains unclear (8, 9).

Like other herpesviruses, HHV-6 has developed immunomodulatory strategies for its life-long persistence in the host's organism (10). HHV-6 replicates in various immune cell types, mainly in CD4 + T-cells and causes a suppression of the immune system (11). Despite various immune-invasive strategies, the healthy immune system is capable to control the virus reactivation during its life-long persistence.

For the closely related human cytomegalovirus (HCMV), the cellular response of CD4+ and CD8+ T-cells has been shown to be fundamental for the control of primary infection and reactivation (12, 13). The aim of this study was to describe HHV-6 specific T-cell immunity in healthy children and adolescents.

## Materials and methods

### Clinical samples

For this prospective, cross-sectional study, peripheral venous blood was drawn from children and adolescents without any inflammatory, immunological or infectious disease before elective surgery for orthopedic pathologies, implant removals, herniotomies, circumcisions, and others. This study was approved by the Institutional Review Board of the Medical University Graz and patients, parents or legal guardians of patients gave written informed consent in accordance with the Declaration of Helsinki.

### Peripheral blood mononuclear cells isolation

Peripheral blood mononuclear cells (PBMCs) were isolated by Ficoll (Ficoll® Paque Plus, GE Healthcare, UK) density gradient centrifugation and were washed twice with DPBS (DPBS, Thermo Fisher Scientific, Carlsbad, California). Trypan Blue (STEMCELL Technologies, 0.4% (m/v), Cologne, Germany) staining was used for the discrimination between viable and non-viable cells and cell numbers were determined via Neubauer chamber.

### Cell culture and stimulation assays

Two different attempts for cell stimulation (overnight and 10 days stimulation) were performed: For the overnight stimulation, PBMCs were suspended in TexMACS medium (Miltenyi Biotec GmbH, Bergisch-Gladbach, Germany). Per well, 1.25^*^10^6^ cells were suspended in 250 μl TexMACS medium in a 48-well plate and were stimulated with mixtures of overlapping 15-mer peptides that encompass the whole protein from U54 (transactivator protein) (PepMix U54, JPT, Berlin, Germany), pp65 (PepMix HCMVA pp65, JPT, Berlin, Germany), and Actin (PepMix Human Actin, JPT; negative control) at a final concentration of 10 μg/ml. On the next day, staphylococcal enterotoxin B (Sigma, Taufkirchen, Germany; positive control, SEB) was added at a final concentration of 2.5 μg/ml to an unstimulated well. Brefeldin A (Sigma, Taufkirchen, Germany) was added as a Golgi stop with a final concentration of 2.5 μg/ml to every well for the last 4 h of incubation before staining.

For 10 days stimulation, PBMCs were suspended in TexMACS medium (Miltenyi Biotec GmbH, Bergisch-Gladbach, Germany). 1.25^*^10^6^ cells were suspended in wells of a 48-well plate and were stimulated with the same antigens as for the overnight stimulation except SEB and pp65 as described above. Cells then were expanded for 10 days in cell culture with interleukin-2 (IL-2; 10 U/mL; Cellgenix, Freiburg, Germany) and interleukin-7 (IL-7; 10 ng/mL; Cellgenix, Freiburg, Germany). Medium was changed every or every second day. On day 9 cytokines were washed out and on day 10 cells were restimulated for 6 h with the same antigens as on day 1 (U54 and Actin). In addition, a previously unstimulated well was stimulated with staphylococcal enterotoxin B (Sigma, Taufkirchen, Germany; positive control). Before staining, Brefeldin A (Sigma, Taufkirchen, Germany) was added to all wells as a Golgi stop for the last 4 h of incubation.

### Flow cytometry staining

PBMCs were then surface-stained with the following monoclonal antibodies to: CD3 (PerCP, Bio Legend, San Diego, USA), CD4 (Alexa Flour 700, Becton Dickinson, San Diego, CA, USA), CD8 (APC-H7, Becton Dickinson, San Diego, CA, USA), CD56 (FITC Becton Dickinson, San Diego, CA, USA). Next, monoclonal antibodies were washed out and stained for live/dead discrimination with an aminereactive dye (Alexa Fluor 350 carboxylic acid, succinimidyl ester, Invitrogen, Carlsbad, CA, USA). After 10 min of incubation at room temperature, cells were fixed and permeabilized using a fix and perm kit® (An Der Grub Bio Research GmbH, Vienna, Austria). Afterwards, intracellular staining was conducted with antibodies against interferon-γ (IFN-γ, BV605 Becton Dickinson, San Diego, CA, USA), interleukin-2 (IL-2, BV510 Becton Dickinson, San Diego, CA, USA) and tumor necrosis factor-α (TNF-α, BV421 Becton Dickinson, San Diego, CA, USA). Measurements were carried out on a flow cytometer LSR II (Becton Dickinson, San Diego, CA, USA) and FACSDIVA acquisition/analysis software (Becton Dickinson, San Diego, CA, USA) was used for data analysis.

### Statistical analysis

The Kruskal-Wallis test and the Fisher's exact test were used to describe the differences in the antigen-specific responses between the two age groups (< 10 years and 10–18 years). Spearman rank correlation was used for describing the degree of association between age and frequency of HHV-6 specific T-cells. *P*-values < 0.05 were considered statistically significant. All statistical evaluations, linear regression, and correlation analyses were performed by SPSS version 23 for Windows (SPSS, Inc., Chicago, IL, USA).

**Figure 1:**
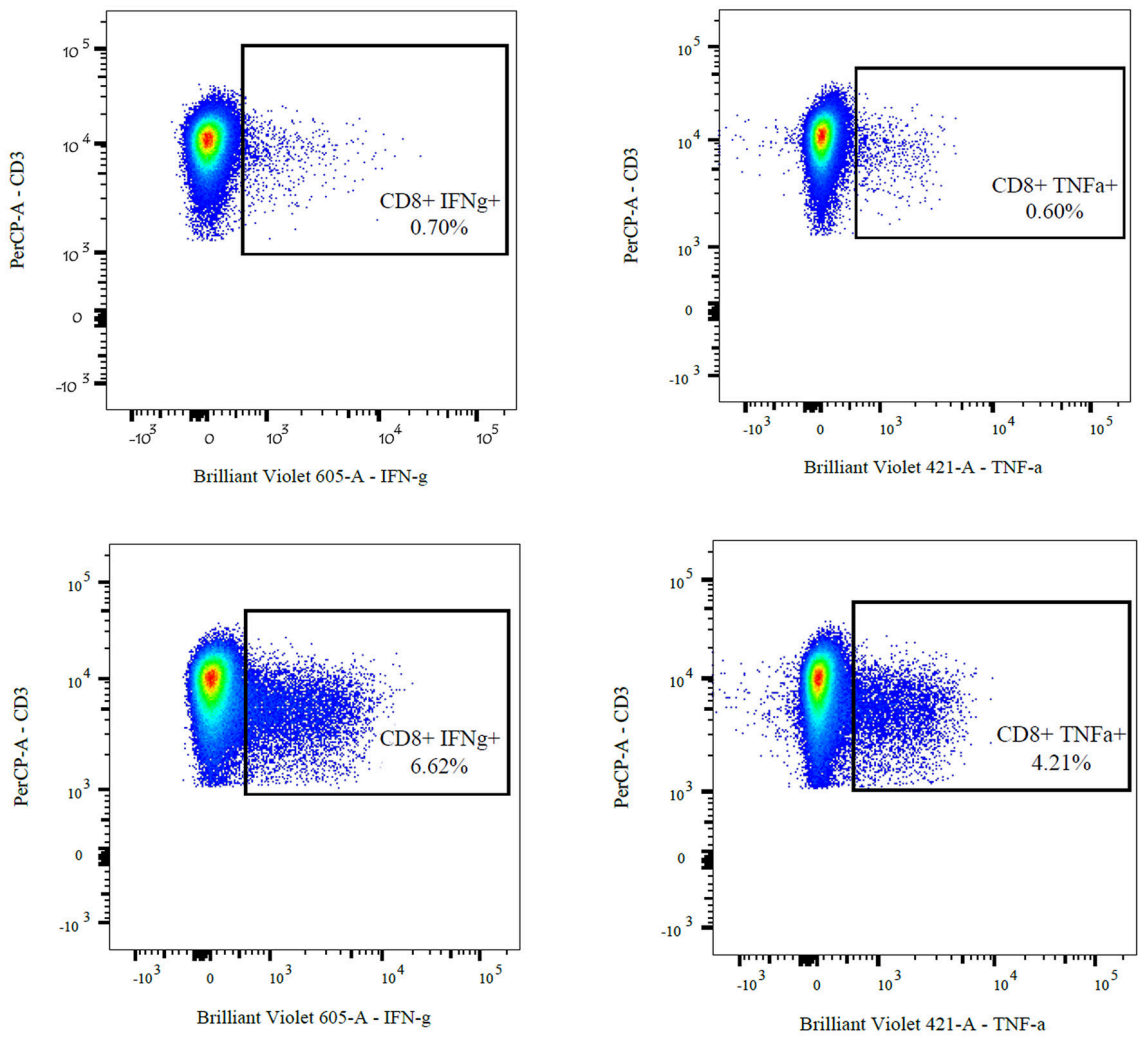
FACS analysis of cytokine-producing CD8 + T-cells of a 17-year-old adolescent after stimulation and 10 days of expansion in cell culture. The upper two images show the negative controls (ACTS) compared to the U54 stimulated cells (**left**: IFN-γ, **right**: TNF-α).

To define the HHV-6 (U54) specific T- cell response, the following 2 criteria had to be fulfilled:

(I) The frequency of cytokine-secreting cells after U54 stimulation in the total population (CD4+ or CD8+ T-cells) was at least 0.1 percentage points higher than in unstimulated cells of the negative control.

(II) The frequency of cytokine-secreting cells after U54 stimulation was at least two times higher than in the negative control (see Figure 1).

## Results

### Short term stimulation

The short overnight stimulation in three individuals (5.7, 8.3, and 9.8 years) with tegument phosphoprotein pp65 showed a CMV-specific immune response in all 3 patients (IFN- γ positive in 1.55, 1.59, and 3.21% of CD4+ T-cells and 1.91, 2.03, and 3.44% of CD8+ T-cells, IL-2 positive in 1.22, 1.71, and 3.41% of CD4+ T-cells and 1.0, 1.54, and 1.93 of CD8+ T-cells, TNF-α positive in 3.11, 3.61, and 3.75% of CD4+ T-cells and 2.6, 3.44, and 3.69 of CD8+ T-cells). In contrast, no HHV-6-specific immune response was detected after stimulation with U54 in none of the tested individuals. For this reason, the stimulation and expansion of the cells was carried out for 10 days in culture for further analyses.

### Long term stimulation (10 days)

In these, 25 healthy children and adolescents were included: 72% male, 28% female, median age 8.2 (range 3.1–18.3) years. All individuals showed an U54-specific response for at least one cytokine in either CD4+ or CD8+ T-cells. Twenty-four percentages of all individuals had a positive response in both cell types. Sixty-eight percentages of individuals had a CD8+ T-cell response for every cytokine. In Table 1, number (percentages) of individuals with U54-specific T-cell responses are shown for different cytokines and cell types.

**Table 1:** Number (percentages) of individuals with U54-specific T-cell response, frequencies of CD4+ and CD8+ T-cells after stimulation with ACTS (negative control), frequencies of CD4+, and CD8+ T-cells after stimulation with SEB (positive control), frequencies of U54-specific CD4+, and CD8+ T-cells and correlation coefficients between age and frequency of HHV-6 specific T-cells (Spearman Rho).

		**Positive/tested individuals (%)**	**Frequency (%) of unstimulated CD4+ and CD8+ T-cells (ACTS) of positive individuals, median (range)**	**Frequency (%) of SEB stimulated CD4+ and CD8+ T-cells (positive control), median (range)**	**Frequency (%) of U54-specific CD4+ and CD8+ T-cells, median (range)**	Correlation coefficient (between age and frequency of HHV-6 specific T-cells) bold ^*^p < 0.05
CD4+	Positive INF-γ response	8/25 (32%)	0.25 (0.1–0.85)	3.83 (0.99–18.07)	1.31 (0.63–10.54)	−0.037
	Positive IL-2 response	3/25 (12%)	0.13 (0.01–0.6)	6.43 (2.06–15)	1.38 (0.10–11.48)	−0.002
	Positive TNF-α response	12/25 (48%)	0.24 (0.06–1.35)	14.45 (5.03–26.80)	2.29 (0.50–24.91)	0.145
	Positive response (any cytokine)	14/25 (56%)	0.5 (0.15–1.95)	17.38 (6.79–29.69)	1.48 (0.12–8.26)	0.168
	Positive IFN- γ/IL-2 response	2/25 (8%)	0.04 (0–0.17)	1.33 (0.29–8.5)	0.05 (0.01–0.29)	0.112
	Positive IFN-γ/TNF-α response	8/25 (32%)	0.07 (0.01–0.55)	2.63 (0.75–15.51)	0.15 (0.01–1.13)	0.077
	Positive IL-2/TNF- α response	3/25 (12%)	0.07 (0–0.25)	4.62 (1.49–11.85)	0.10 (0–0.30)	−0.079
	Positive IL-2/TNF-α/IFN-γ response	2/25 (8%)	0.03 (0–0.17)	1.16 (0.28–8.2)	0.05 (0.01–0.25)	−0.018
CD8+	Positive INF-γ response	10/25 (40%)	0.33 (0.13–1.05)	7.25 (2.03–18.49)	1.69 (0.80–10.21)	0.088
	Positive IL-2 response	3/25 (12%)	0.12 (0–0.55)	2.35 (0.31–4.92)	1.01 (0.60–4.67)	0.225
	Positive TNF-α response	14/25 (56%)	0.34 (0.05–0.8)	7.02 (2.49–12.89)	2.10 (0.72–9.83)	0.465^*^
	Positive response (any cytokine)	17/25 (68%)	0.59 (0.18–1.35)	10.99 (4.58–21.29)	1.86 (0.69–8.04)	0.286
	Positive IFN-γ/IL-2 response	2/25 (8%)	0.04 (0–1.15)	0.49 (0.17–2.57)	0.07 (0–0.20)	0.146
	Positive IFN-γ/TNF-α response	11/25 (44%)	0.13 (0.02–0.42)	4.2 (1.1–10.32)	0.23 (0.02–2.92)	0.142
	Positive IL-2/TNF-α response	1/25 (4%)	0.05 (0–0.25)	0.67 (0.06–2.39)	0.07 (0–0.34)	0.141
	Positive IL-2/TNF-α/IFN-γ	1/25 (4%)	0.03 (0–0.15)	0.37 (0.06–2.15)	0.05 (0–0.2)	0.083

IFN-γ, interferon-γ; IL-2, interleukin-2; tumor necrosis factor-α, TNF-α. The bold value marks the positive correlation coefficient of r = 0.465 and the

* *refers to the significance level of p < 0.05*.

Percentages of individuals with HHV-6-specific TNF-α response were the highest for CD4+ (48% of individuals) as well as for CD8+ (56% of individuals) and TNF-α secreting CD4+ cells were the most prominent HHV-6-specific cells. Noteworthy, only 12% of individuals showed a positive IL-2 response in both CD4+ and CD8+ cells, while IL-2 response was clearly positive after stimulation with SEB (positive control) proofing reliable intracellular staining with the used antibody (see Table 1).

**Figure 2:**
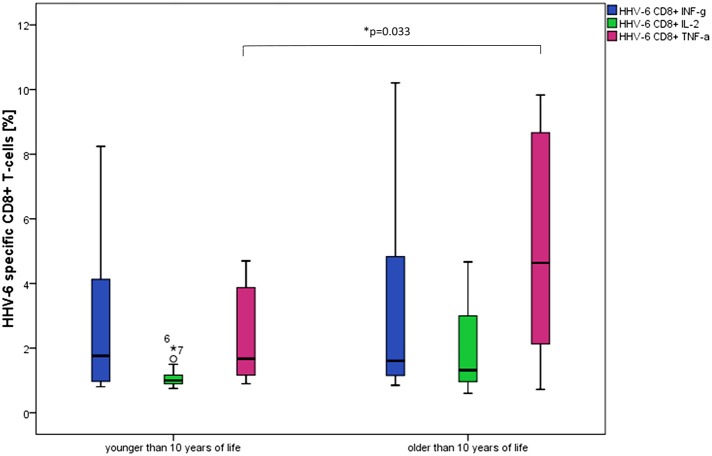
Box plots show significantly higher frequencies of TNF-α producing CD8+ T-cells in individuals older than 10 years after stimulation with HHV-6 specific antigen U54. ^*^p = 0.033. Differences between the age groups were not statistically significant for the other cytokine patterns and CD4+ T-cells.

Comparing the age groups (<10 years, *n* = 13 and 10–18 years, *n* = 12), significantly higher frequencies of HHV-6-specific TNF-α producing CD8+ T-cells in the older age group (*p* = 0.033) were detected [<10 years, median age 6.6 (range 3.1–9.2) and 10–18 years, median age 17.2 (range 10.5–18.3)] (see Figure 2).

**Figure 3:**
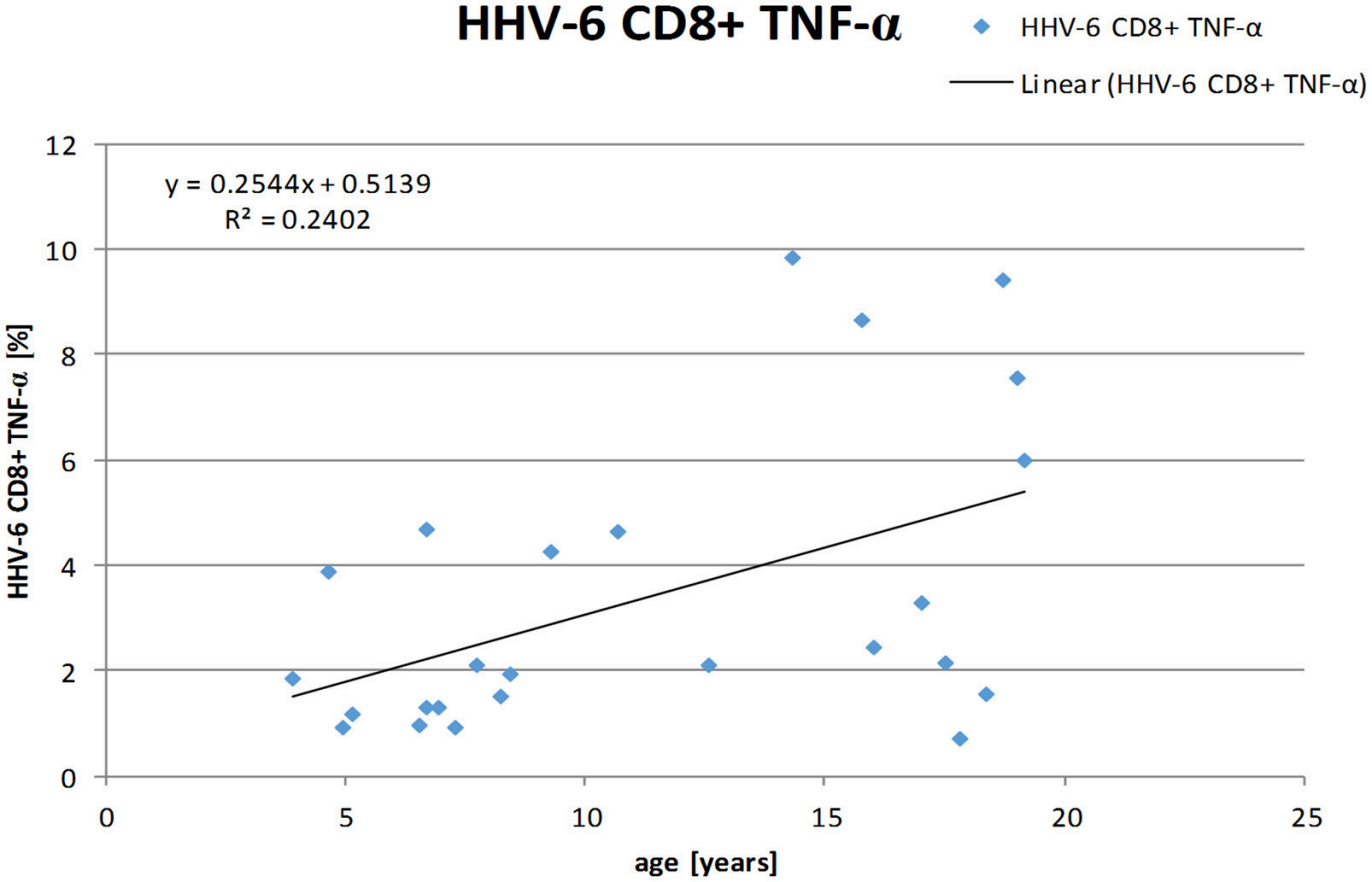
Linear regression analysis shows a positive correlation between age and frequencies of HHV-6-specific TNF-α producing CD8+ T-cells. (Spearman-Rho *r* = 0.490).

The frequency of TNF-α producing CD8+ T-cells positively correlated with the age of the individuals *r* = 0.465 (Spearman-Rho) on a two-sided significance level of *p* = 0.019 and linear regression analysis showed a positive relation between age and frequency of HHV-6-specific TNF-α producing CD8+ T-cells with a regression coefficient of *r* = 0.490 (see Figure 3). No positive correlations were found for the other cytokines.

## Discussion

After primary infection, human herpes viruses establish a lifelong latent infection in the host and persist in certain host cells. Latently infected cells may express virus-specific antigens on their cell surface (15, 17). However, to protect themselves from immuno-recognition, human herpes viruses have a limited viral gene expression during latency (13). Subsets of T-cells that have specific cytotoxic activity against virus-infected or transformed cells are crucial for the suppression of reactivation (16, 17).

We, therefore, analyzed secretion of IL-2, IFN-γ as well as TNF-α in both CD4+ and CD8+ T-cells after stimulation with the HHV-6 specific antigen U54 and demonstrated the presence of HHV-6-reactive CD4+ and CD8+ T-cells in the peripheral blood of healthy children and adolescents. We have chosen a peptide mix derived from U54, which is a tegument protein of HHV-6 and the analog of the CMV lower matrix phosphoprotein 65 (pp65). The pp65 protein is one of the most dominant CD4+ and CD8+ T-cell antigens of CMV and is therefore widely used for the evaluation of CMV-specific immunity (14).

In a first set of experiments, we tested a short overnight stimulation of PBMCs with U54 antigen, but we were not able to reliably detect an antigen-specific response. A short-term stimulation reflects the *in vivo* situation more accurately but the *in vivo* frequencies of U54 specific T-cells were too low to be detected by flow cytometry. Consequently, we have decided to culture the cells in medium and interleukins for 10 days to obtain an adequate antigen-specific immune response against U54 (12).

For expansion of antigen-specific T-cells we used the cytokines IL-2 and IL-7. IL-7 promotes the proliferation of immature naïve human T-cells that have recently exited from the thymus (11, 12). IL-7 together with IL-2 primes naïve peripheral blood T-cells as well as recently exited T-cells for Fas (First apoptotic signal)-mediated cell death. However, IL-2/IL-7-stimulated memory T-cells are significantly more resistant to Fas-induced cell death (13). It must be considered that IL-2 and IL-7 promote homeostatic expansion of memory T-cells while limiting the size of the overall T-cell pool.

After stimulation, 10 days of cultivation and re-stimulation of PBMCs, an U54-specific response was observed in all individuals proofing the *in vivo* presence of HHV-6 specific T-cells capable of expansion after adequate *in vitro* stimulation. It might be assumed that these cells would also expand *in vivo* in clinical conditions necessitating increased immunological response. Individuals older than 10 years had significantly higher frequencies of HHV-6 specific CD8 + TNF-α T-cells than those younger than 10 years of age and data analysis showed a positive correlation between age of the individuals and frequency of these virus specific T-cells. In further studies, cytotoxicity assays should analyze the functional impact of these observed numeric differences.

Our results are in line with the fact that immunity to HHV-6 could evolve over time. Commonly, neonates are protected from HHV-6 infection by maternal-derived antibodies up to 3–9 months postnatally. A primary infection generally occurs before the second year of life leading to the persistence of viruses and latent infection with HHV-6, and in most cases antibodies persist throughout life (17). In parallel, the development of the HHV-6-specific T-cell response after primary infection must be assumed with an evolution of this response throughout life, driven by the interplay between latent infection and immunological response. This might be reflected by the observed higher CD8+ TNF-α T-cell frequencies in older children. In the current study, we did not perform serial testing of antigen-specific immunity to HHV-6 longitudinally but it would be interesting in future studies to test how cellular immunity against HHV-6 develops within single individuals.

HHV-6 specific T-cells play a key role in controlling the virus infection and overcoming immunomodulatory effects of HHV-6. The majority of expanded effector CD4+ and CD8+ populations secrete IFN-γ and TNF-α and have various cytolytic functions, mediated by the secretion of perforin (CD4+) or granzyme B (CD8+). These T-cells can rapidly lyse infected cells and reacquire cytotoxic activity when they are exposed to the antigen (20).

Interestingly, we could only detect few antigen-specific IL-2 producing T-cells. This might be explained by the fact that after restimulation with the U54 antigen both central memory T-cells as well as effector memory T-cells produce high amounts of TNF-α and IFN-γ but IL-2 is mainly produced by central memory cells (21, 22). HHV-6 serology was not tested due to the fact that nearly 100% of individuals older than 2 years of life are seropositive (7). For the same reason, we were not able to include HHV-6 seronegative individuals as negative controls.

There have been a limited number of studies on the specificity of the immune response to HHV-6, although cellular immune responses are fundamental in controlling primary infection and reactivation of the virus. Some earlier studies investigated T-cell proliferative responses to HHV-6 in healthy adults (15, 16). Yakushijin et al. generated CD4+ T-cell clones from HHV-6-stimulated PBMC that were found to proliferate upon stimulation with HHV-6 in the presence of autologous antigen-presenting cells and Yasukawa et al. investigated on the antigenic specifity of CD4+ T-cell clones against HHV-6, HHV-7, and HCMV. However, there are no detailed studies on the cellular immune response in healthy children and adolescents. Interestingly, Koide et al. described an immature lymphocytic response to HHV-6 antigen in children below 2 years (17). This is in line with our results and indicates that the virus-specific immune response after primary infection develops over time.

In individuals with chromosomal integrated HHV-6, antigen-specific T-cells have also been measured by Enzyme Linked Immuno Spot Assay (18).

Virus specific T-cells are also important for patients during immune reconstitution after hematopoietic stem cell transplantation (HSCT). Studies have shown that donor-derived virus-specific T-cells against different viruses were administered with impressive clinical results (19–24). These donor-derived virus- specific T-cells may constitute a cost-effective and more effective alternative to common antiviral agents for allogeneic HSCT recipients but requires a detailed understanding of virus-specific T-cells. Our results are intended to contribute to this knowledge. Furthermore, our results derived from healthy individuals will help to assess and interpret HHV-6 specific T-cell response in selected patient groups at higher risk for complicated HHV-6 infection or reactivation like recipients of HSCT.

To conclude, in our study we tested antigen-specific T-cell immunity to HHV-6 in healthy individuals. HHV-6-specific T-cells can be expanded in 9-day culture with addition of the proliferative cytokines IL-2 and IL-7 and in response to the viral antigen (U54) for analyses. Further studies on HHV-6-specific T-cell immunity in healthy individuals and in patients with impaired immune system should shed more light on the mentioned interplay and the immunopathology of HHV-6 infection and control of the virus.

## Data availability statement

The raw data supporting the conclusions of this manuscript will be made available by the authors, without undue reservation, to any qualified researcher.

## Author contributions

CS, VS, HS, WS, and CU designed the research study. CS and AR conducted the experiments and acquired data. CS and VS analyzed data. GS and MK provided samples. CS, VS, HS, and GS wrote the manuscript.

### Conflict of interest statement

The authors declare that the research was conducted in the absence of any commercial or financial relationships that could be construed as a potential conflict of interest.
